# Genomic and cytogenetic evidence for divergence and speciation in geladas

**DOI:** 10.1186/s13059-026-04143-6

**Published:** 2026-07-22

**Authors:** Brooklynn R. Scott, Ferehiwot Ayele, Alemayehu Lemma, Amera Moges, Belayneh Abebe, Ashlee Greenier, Elizabeth Slikas, Michael Blackwell, Kerstin Becker, Megan Stoltzmann, Baptiste Sadoughi, Achim Johann, Nicolas Brüning, Abebaw Azanaw Haile, Fanuel Kebede, Fekede Regassa Joka, Andrea Guarracino, Addisu Mekonnen, Allyson Hindle, Jacinta C. Beehner, India A. Schneider-Crease, Amy Lu, Thore J. Bergman, Kenneth L. Chiou, Noah Snyder-Mackler

**Affiliations:** 1 School of Life Sciences, Arizona State University, Tempe, AZ USA; 2 Center for Evolution and Medicine, Arizona State University, Tempe, AZ USA; 3 College of Veterinary Medicine and Agriculture, Addis Ababa University, Addis Ababa, Ethiopia; 4 Department of Biology, College of Science, Bahir Dar University, Bahir Dar, Ethiopia; 5 School for Human Evolution and Social Change, Arizona State University, Tempe, AZ USA; 6 School of Life Sciences, University of Nevada, Las Vegas, NV USA; 7 Cologne Center for Genomics, Medical Faculty, University of Cologne, Cologne, Germany; 8 NaturZoo Rheine, Rheine, Germany; 9 Ethiopian Wildlife Conservation Authority, Addis Ababa, Ethiopia; 10 Bioinnovation and Genome Sciences, The Translational Genomics Research Institute, Phoenix, AZ USA; 11 Department of Anthropology and Archaeology, University of Calgary, Calgary, Canada; 12 Department of Psychology, University of Michigan, Michigan, MI USA; 13 Department of Anthropology, Stony Brook University, Stony Brook, NY USA; 14 Department of Biology, University of Alabama at Birmingham, Birmingham, AL USA; 15 ASU-Banner Neurodegenerative Disease Research Center, Arizona State University, Tempe, AZ USA

## Abstract

**Background:**

Chromosomal rearrangements can play an important role in shaping patterns of genomic divergence and speciation, yet their evolutionary context is still largely unclear, as such events are rare and challenging to identify in recent evolutionary history. Geladas (*Theropithecus gelada*) provide a unique system to investigate this process, as a novel fission on chromosome 7 has resulted in polymorphic karyotypes among gelada populations.

**Results:**

We integrate new cytogenetic data from ten animals together with whole-genome sequencing from 149 individuals spanning all three extant gelada populations (northern, central, and southern). We report additional cytogenetic evidence consistent with fixation of a chromosomal fission on chromosome 7 in northern geladas, and the absence of the fission in the central population. Phylogenetic analyses reveal that the southern lineage diverged first, prior to the split between northern and central geladas, strongly suggesting that the southern population retains the ancestral karyotype. Demographic modeling further refines the timing of the northern–central split, indicating a more recent divergence (~ 160 thousand years ago) than previously estimated. We detect elevated genetic differentiation between the northern and central populations and reduced nucleotide diversity in the northern lineage on chromosome 7, which is consistent with historically suppressed recombination. These results suggest that the fission on chromosome 7 may have contributed to regional divergence by promoting recombination suppression in heterokaryotypes.

**Conclusions:**

By combining cytogenetics with population genomes, these findings highlight the role of chromosomal evolution in shaping localized genomic differentiation, and its potential for reinforcing reproductive isolation. Our results also suggest that northern geladas may represent an incipient species.

**Supplementary Information:**

The online version contains supplementary material available at https://doi.org/10.1186/s13059-026-04143-6.

## Background

Understanding the mechanisms that drive speciation is essential for explaining patterns of biodiversity and adaptation. Chromosomal rearrangements have been hypothesized to restrict gene flow and promote reproductive isolation among populations, thereby shaping the speciation process [1]. Chromosomal fissions and fusions, where chromosomes break apart (fission) or merge (fusion), are common drivers of interspecific karyotype diversity in mammals and other taxa. Yet, the extent to which chromosomal rearrangements directly drive speciation are currently unknown [1–3] because intraspecific chromosomal polymorphisms are exceptionally rare [4–6].

Additionally, the rate of karyotype evolution differs greatly between species, raising fundamental questions about species- and lineage-specific tolerance for chromosomal change [2]. While karyotype flexibility may enhance genetic diversity and adaptability in some taxonomic groups, it could reinforce reproductive isolation in others. Addressing this gap in primates is especially important given the potential for producing comparative insights on how chromosomal evolution has shaped human evolutionary history.

**Fig. 1:**
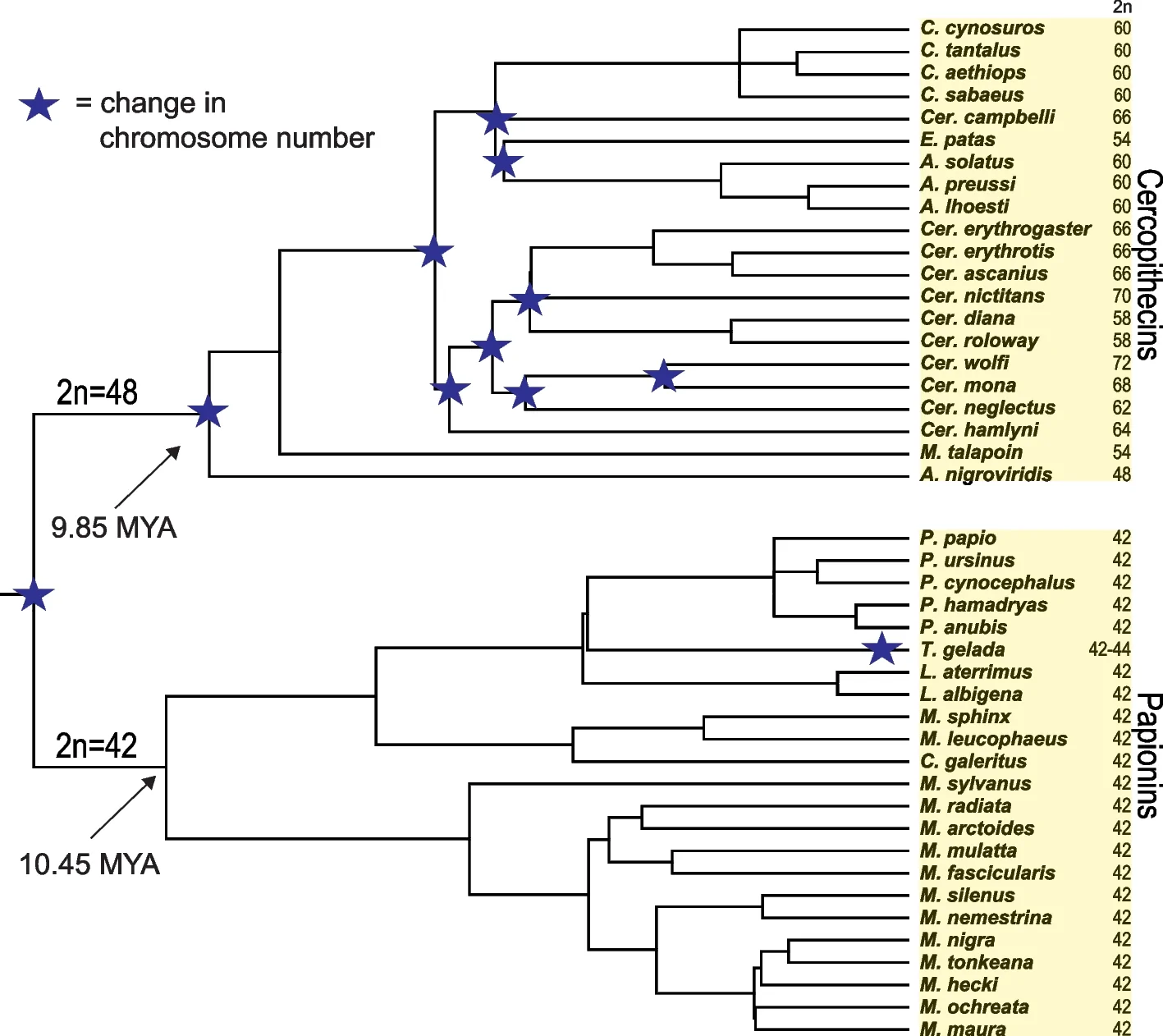
Chromosomal variation in papionins and cercopithecins: Geladas are the only member of the papionini clade that does not have the conserved karyotype of 42 diploid chromosomes. This contrasts with their sister tribe, the cercopithecins, which show a more variable karyotype ranging from 48–72 diploid chromosomes. Karyotype data was sampled from Stanyon et al. [8] and Moulin et al. [9] and are shown with the consensus chronogram from TimeTree [10]. Recent genome-wide phylogenies [11] provide updated resolution of relationships within Cercopithecini but do not alter the overall pattern of marked karyotypic dynamism in this clade relative to Papionini

The cercopithecine subfamily presents an opportunity to explore the dynamics of chromosomal change. The clade includes two major tribes, Papionini (“papionins”: baboons, macaques, and their relatives) and Cercopithecini (“cercopithecins”: guenons, vervets, and their relatives), which show striking contrast in rates of karyotypic evolution (Fig. 1). Papionins are characterized by extreme chromosomal stability, while cercopithecins have exceptionally high rates of karyotype evolution, with chromosome numbers ranging from 48 to 72 [7]. This contrast creates a natural comparative system in which closely related lineages display markedly different rates of karyotype evolution. Most primate studies to date have focused on cytogenetics, with relatively few leveraging the power of modern genomic data. Incorporation of genomic data can help pinpoint how and when rearrangements occur and, combined with cytogenetics, provide a more complete picture of chromosomal change.

**Fig. 2:**
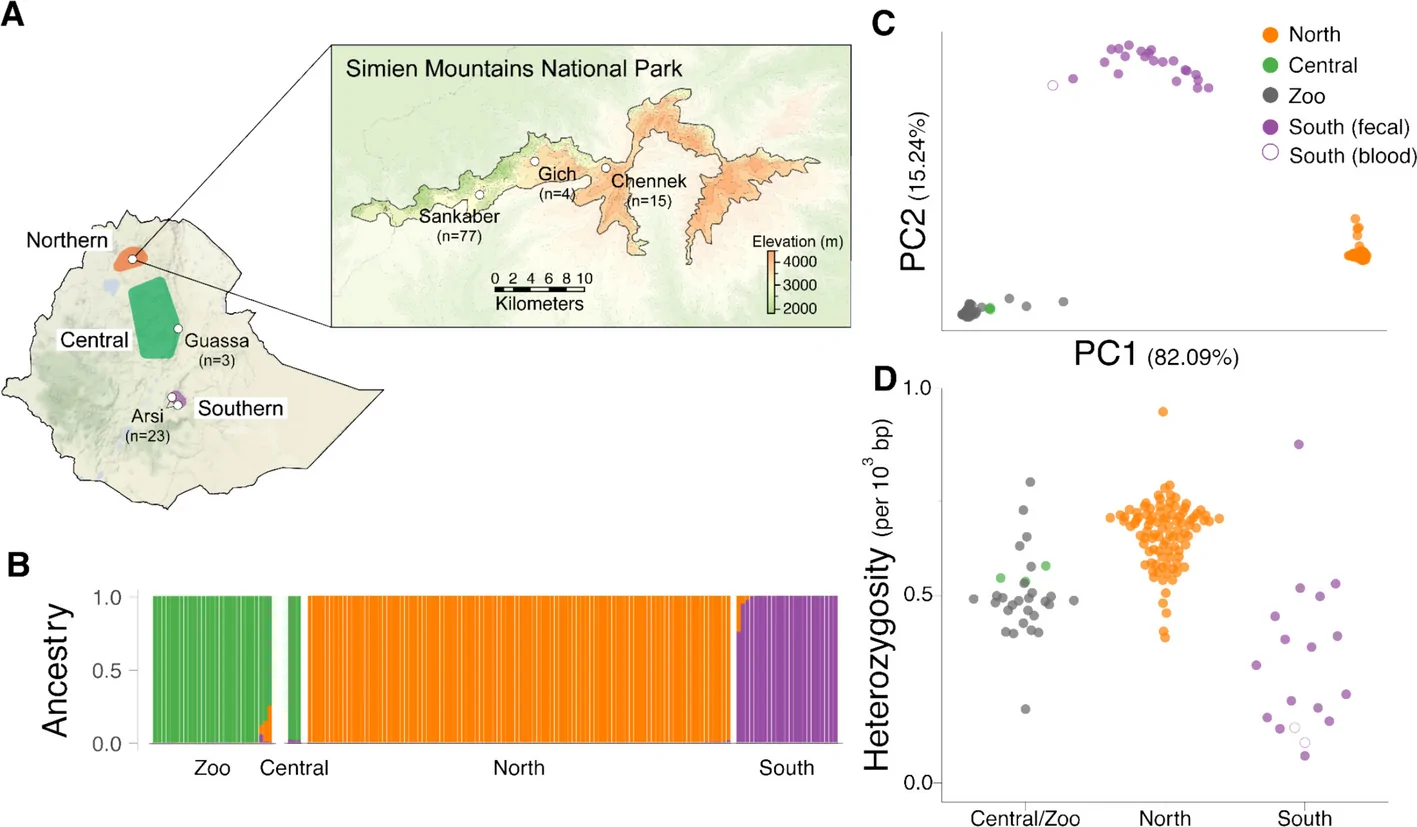
Summary of genomic dataset, population structure, and genetic diversity in geladas: **A** Geladas form three main populations across Ethiopia. Points indicate sampling locations, with numbers corresponding to individuals sampled at each site. **B** NGSadmix plot showing ancestry proportions for K = 3 from 149 geladas reveals three main populations: northern (orange), central (green), and southern (purple). Zoo animals are primarily central ancestry. The two animals with the highest proportion of northern ancestry are heterozygous for the chromosomal fission. **C** Principal component analysis separates samples into three main clusters corresponding to the northern (orange), central (green), and southern (purple) populations. Southern blood samples from Caldon et al. [17] are shown with open circles. **D** Per-site heterozygosity shows substantially lower values in the southern population relative to northern and central populations. Note that five southern fecal samples with heterozygosity > 0.001 are not shown, as these are likely lower quality or reflect cross-contamination

Among papionins, geladas (*Theropithecus gelada*) represent a significant outlier in their karyotypic evolution. Geladas are restricted to high-altitude grasslands of Ethiopia (~ 1800–4500 m.a.s.l.) and are geographically divided into three population groups, sometimes considered subspecies–northern (*T. gelada gelada*), central (*T. gelada obscurus*), and southern (*T. gelada arsi*) [12, 13] (Fig. 2A). All three populations are separated by valleys that form natural barriers among them. After diverging from *Papio* baboons and *Lophocebus* mangabeys approximately 5–7 million years ago [11, 14], a chromosomal fission of chromosome 7 gave rise to a derived karyotype of 2n = 44 in geladas that was first observed in the northern population [15]. This differs from the ancestral karyotype 2n = 42 seen in all other papionin species, including in most zoo-born geladas [7, 8, 15, 16]. The fission on chromosome 7 has led to the formation of two new chromosomes that persist alongside the ancestral intact chromosome in other geladas, marking a significant departure from the otherwise stable karyotype within the clade. Little is known about the demographic history of the three gelada populations, or the extent and distribution of the chromosome 7 rearrangement. These unique properties make geladas a rare and powerful model for studying intraspecies chromosomal evolution in a lineage that typically resists such change [15].

Given the expected impact of a change in chromosome number, the fission has likely played an important role in gelada evolutionary history, yet its origins and consequences remain unknown. Previous work suggested the fission may be fixed in the northern population [15], but cytogenetic data was limited. How quickly it became fixed and how this process relates to population divergence remain unclear. To address this, we combine new karyotype data with expanded whole-genome sequencing from all three populations. We use this data to place the chromosome 7 fission within a clarified demographic framework which allows us to infer both when this rearrangement likely arose and how it may have contributed to broader patterns of population divergence. In addition to refining the branching relationships among northern, central, and southern geladas, we assess whether the rearranged chromosome exhibits signatures of elevated genetic differentiation and lineage-specific reductions in nucleotide diversity. These analyses allow us to test whether the fission is associated with localized genomic divergence consistent with altered recombination dynamics. ​​Importantly, our novel genomic data from the southern population, which has historically been underrepresented in comparative work, provide critical context for testing whether the fission is a derived feature of northern geladas or whether it reflects deeper ancestry.

## Results

### Genetic diversity and population structure

To better understand the population structure of geladas, we used whole-genome sequencing (WGS) data from 149 individuals across all three populations in Ethiopia (*N* = 96 wild northern; *N* = 3 wild central; *N* = 23 wild southern; and *N* = 27 captive geladas with central ancestry) (NCBI Bioproject: PRJNA470999) [18, 19]. The 96 northern animals are further divided into three subpopulations–Sankaber (*N* = 77), Gich (*N* = 4), and Chennek (*N* = 15) (Fig. 2A).

Our dataset substantially expands available genomic resources. We include 46 newly sampled individuals from the northern population (mean depth = 7.2x) and 8 newly sampled individuals with central ancestry from NaturZoo Rheine (mean depth = 9.0x), all of which were sequenced from blood samples.

Notably, we include newly-generated WGS and cytogenetic data from Gich, part of the northern gelada range, which had not been sequenced in previous studies. We also include new genomic data from 21 wild southern geladas, a population which has not been extensively sampled, representing the largest WGS dataset on southern geladas to date. This population, which occupies the southernmost extent of the species’ range, is the smallest and most vulnerable to habitat loss, making it a high conservation priority. We generated WGS data from fecal material rather than blood in this population due to sampling constraints, which produced lower quality data than blood-derived samples (mean depth = 0.90x) but allowed for the inclusion of data (*n* = 21 individuals) from this understudied population. Lower coverage sequencing data can reduce genotype certainty and limit the power for fine scale analyses, therefore we also include two southern gelada samples from Caldon et al. [17], which were generated from blood (mean depth = 19.67x), providing higher coverage reference data for comparison (NCBI Bioproject: PRJEB72259) [20].

We first carried out two different unsupervised clustering analyses on the 149 WGS samples, both of which supported three distinct populations corresponding to the three geographically-linked subspecies (Fig. 2B, C). Admixture analysis reflected three primary ancestry components (K = 3), corresponding to the northern, central, and southern populations (Fig. 2B). Wild individuals showed minimal evidence of admixture, suggesting historically limited gene flow between populations. Notably, despite the lower coverage and quality, fecal-derived southern WGS samples clustered with the two blood-derived southern samples (Fig. 2C), suggesting that ancestry information is robustly recovered from lower-quality fecal samples [21, 22].

We next quantified heterozygosity for each individual for all populations to assess genetic diversity, which is critical for maintaining population viability and can reflect adaptive potential to changing environments. We found that the southern population had significantly lower heterozygosity relative to both the northern (*p* < 2 × 10^–16^) and central (*p* = 7.0 × 10^–5^) populations, in a linear model controlling for sequencing depth (Fig. 2D; five outlier southern samples were removed based on excessive heterozygosity indicative of cross-contamination). These results suggest that the southern population is at greater risk of inbreeding depression and highlight the urgency of prioritizing conservation measures for southern geladas.

Finally, consistent with prior findings [15], three zoo individuals deviated from the central cluster along PC1 and showed clear signatures of admixture (Fig. 2B and C). Two of these individuals had heterozygous karyotypes (2n = 43), consistent with mixed ancestry. Using previously identified population-specific mitochondrial haplotypes [12] along with northern and central ancestry proportions, we inferred that the two heterozygous individuals are likely F2 and F3 hybrids (Additional file 1: Figure S1). The remaining zoo animals, including all newly sampled individuals, clustered closely with the wild central animals, consistent with the pattern of zoo animals being of central origin.

### Karyotype variation

Because previous work was limited in sample size, additional karyotyping was needed to confirm whether the chromosome 7 fission is fixed across northern gelada subgroups. Our previous work suggested that the novel variant is a uniquely northern trait [15], but this was based on only four karyotyped individuals–most of which were derived from one northern subgroup. Here, we generated an expanded karyotype dataset spanning all three subgroups of the northern population, including four newly sampled individuals from Sankaber, four from Chennek, and two from Gich [23]. Our data are consistent with prior hypotheses: all individuals (*N* = 14) from the northern populations were homozygous for the derived karyotype (2n = 44).

In contrast, all individuals from the central population (*N* = 2), and those with confirmed central ancestry from zoos (*N* = 7), retained the ancestral karyotype (2n = 42) [15] (Table 1). We also identified additional karyotype data from zoo individuals (*N* = 5) for whom direct ancestry assignments were not available [8, 16]. Our genomic analyses indicate that zoo geladas are predominantly of central ancestry, therefore we include them here as central individuals (Table 1).

**Table 1:** Number of karyotyped individuals in each population

Population	Ancestry	Number of individuals karyotyped	Karyotype (2n)
northern (wild)	northern	14 (10 new)	44 (all)
zoo	central	13 (1 new)	42 (all)
zoo	northern-central hybrid	3	43 (*N* = 2); 42 (*N* = 1)
central (wild)	central	2	42 (all)

Total number of individuals karyotyped for each population, including previously published data [8, 15, 16, 24]

This estimated allele frequency of the fissioned chromosome in the central population of 0% (95% CI: 0–9.5%), which was supported by both metaphase spread analyses and Hi-C data, demonstrates a clear pattern of chromosomal divergence and are consistent with the absence of the chromosomal fission in the central population.

### Demographic history of geladas

**Fig. 3:**
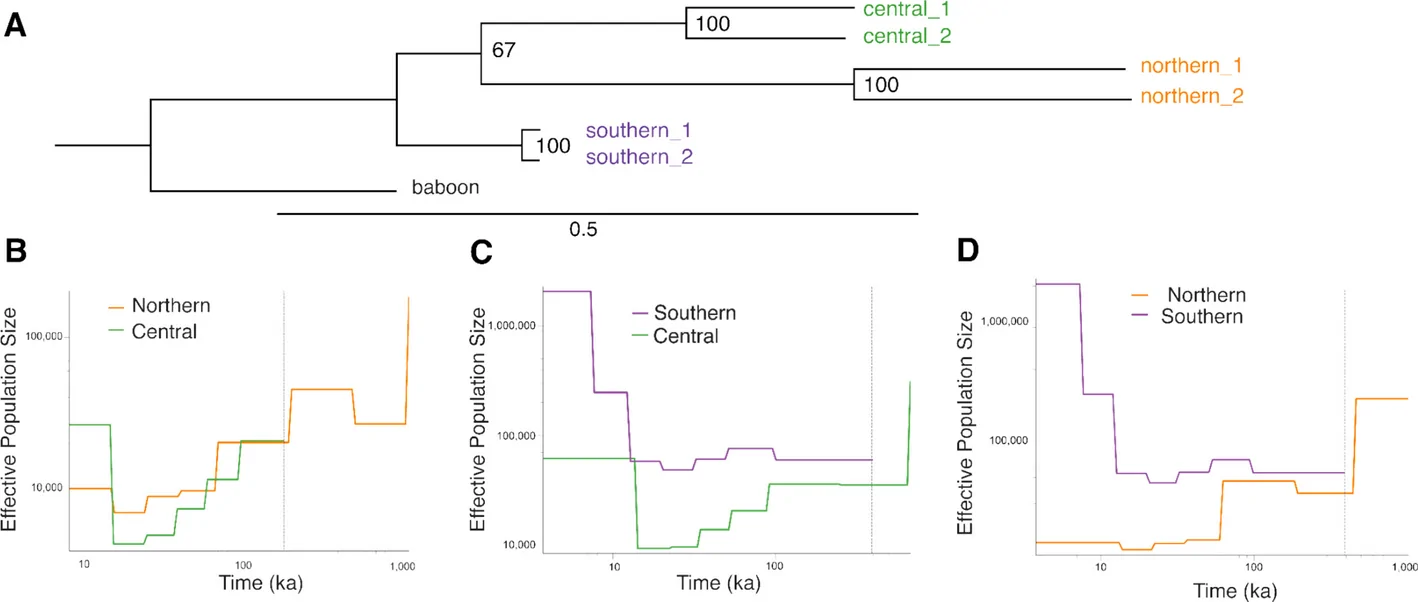
Phylogenetic relationships and demographic history of three gelada populations: **A** Maximum likelihood phylogeny of gelada populations inferred with IQ-TREE [25] using autosomal SNPs from two high-quality individuals per population. Bootstrap support values are shown at all nodes. **B** Population split time estimates between the northern and central gelada populations inferred with SMC++, with an estimated split ~ 157 kya. **C** Population split time estimates between the central and southern gelada populations inferred with SMC++, with an estimated split ~ 393 kya. **D** Population split time estimates between the northern and southern gelada populations inferred with SMC++, with an estimated split ~ 388 kya

Previous mitochondrial and microsatellite analyses have yielded discordant relationships between the three gelada populations [12, 13]. To obtain a genome-wide perspective, we reconstructed a phylogeny using SNP data across all autosomes from two gelada individuals per-population. We also included a single baboon as an outgroup. Using this data we inferred a maximum likelihood tree with IQ-TREE [25] and found support (67% bootstrap) for a tree where the southern population diverges from the ancestral north/central prior to the split between the northern and central populations (Fig. 3A). Reduced support at this node suggests that a fraction of loci support alternative relationships between gelada populations. This uncertainty, observed across mitochondrial, microsatellite, and genome-wide SNP data, is consistent with discordant genealogical histories among genomic regions, as predicted under scenarios of recent speciation [26].

While our phylogenetic analysis offers insight into the relationships between the three gelada populations, it does not resolve the timing of population splits nor the demographic history of each population. To address this, we applied two complementary approaches to estimate the divergence time between the northern and central populations. We also used a single method to estimate divergence between the central or northern population from the southern population, due to limited coverage and a smaller sample size from the southern population. Importantly, these demographic approaches use genome-wide allele frequency patterns rather than relying on a single inferred tree, making them less sensitive to conflicting genealogical signals [27, 28].

To reconstruct the evolutionary relationships and model the demographic history of geladas, we first applied SMC++ [28] to northern-central, central-southern, and northern-southern datasets, providing initial estimates of split times. SMC++ estimated the split between the northern and central populations at ~ 157,000 years ago (Fig. 3B). The split between the southern and central populations (~ 393,000 years ago; Fig. 3C) was similar to the estimated split between the southern and northern populations (~ 388,000 years ago; Fig. 3D). The similarity of these estimates is consistent with a phylogenetic scenario in which the southern population diverged prior to the split between the northern and central populations.

Building on these results, we then used dadi-cli [27] to model two-population demographic scenarios for the northern and central populations, allowing us to refine our understanding of their divergence and demographic history beyond simply estimating the timing of the split. We focused on four two-population models (*Methods*), which were chosen based on prior knowledge about the relationship between these populations.

**Fig. 4:**
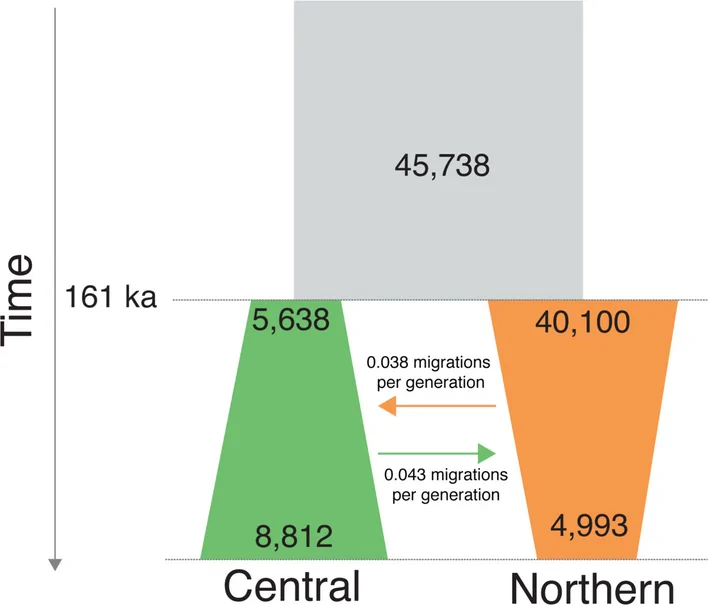
Two population demographic model between northern and central geladas: Best-fit demographic model inferred with *dadi-cli* [27] under an Isolation-with-Migration (IM) framework. The model supports a split between the northern and central populations ~ 13,800 generations ago (~ 161,000 years ago) with low migration rates. Numbers in the model indicate effective population sizes (Ne) for each population

The best fit model was isolation with migration (IM). Under this model, the estimated population split between the northern and central populations occurred ~ 13,800 generations ago (~ 161,059 years ago), with evidence of low migration between the populations (Fig. 4). This estimate is highly concordant with the split time inferred using SMC + +.

### Patterns of genetic differentiation and diversity on the rearranged chromosome 7

Chromosomal rearrangements are predicted to reshape recombination landscapes and, consequently, patterns of linked selection and genomic divergence [1]. To test whether the chromosome 7 fission was associated with localized differentiation beyond genome-wide expectations, we quantified windowed F_st_ and population-specific nucleotide diversity (π) across autosomes (Additional file 1: Figure S2). For subsequent statistical analyses, chromosomes 7a and 7b were treated as a single combined unit (hereafter referred to as “chromosome 7a/7b”).

Chromosome 7a/7b showed an excess of highly differentiated regions. Although chromosome 7a/7b represents a combined 6.33% of autosomal windows, it harbors 11.33% of windows in the upper 1% of the genome-wide F_st_ distribution (1.79-fold enrichment; binomial test, *p* = 8.68 × 10⁻^22^), demonstrating significant enrichment for highly differentiated regions.

**Fig. 5:**
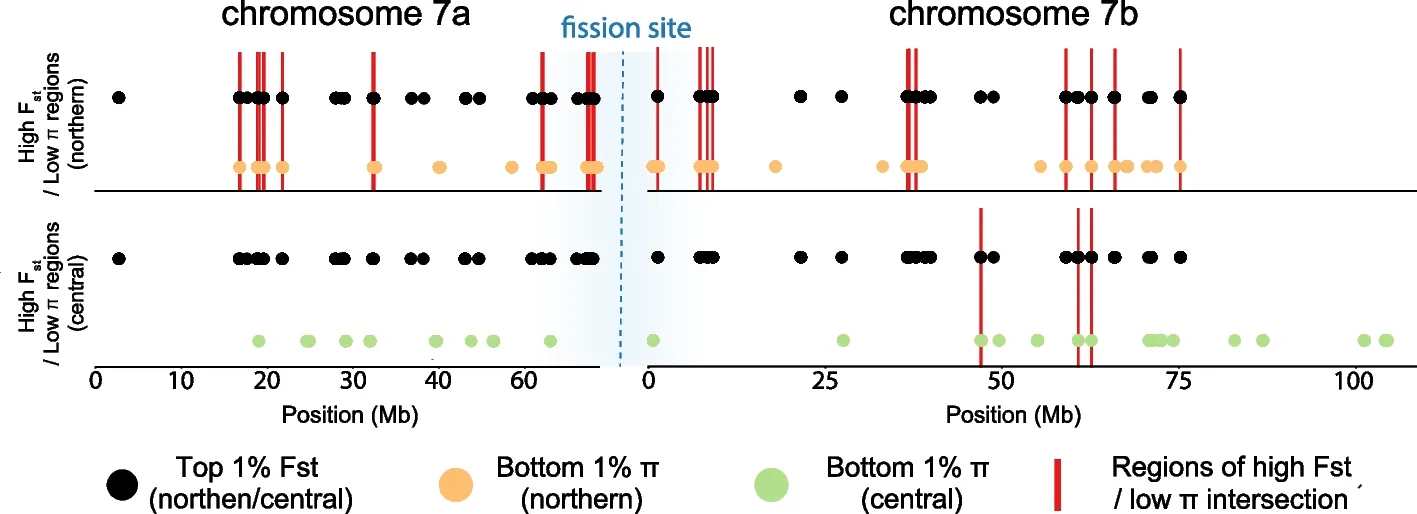
Genomic distribution of highly differentiated (top 1% F_st_) and low-diversity (bottom 1% π) windows along chromosomes 7a and 7b: Windows in the top 1% of the genome-wide F_st_ distribution between northern and central populations are shown in black and are identical in both panels. Bottom 1% π windows are shown separately for the northern population (top panel; orange) and the central population (bottom panel; green). Windows exhibiting both elevated F_st_ and reduced π are highlighted in red. Genomic coordinates are plotted along chromosomes 7a and 7b, with the fission breakpoint indicated

We next tested whether the excess differentiation was accompanied by lineage-specific reductions in nucleotide diversity, as predicted under localized recombination suppression [29]. If the fission caused reduced effective recombination in heterozygotes during its fixation, we would expect an enrichment of windows with reduced π in the northern lineage, but not in the central. Windows in the lowest 1% of the northern π distribution were strongly enriched on chromosome 7a/7b (12.89% observed vs. 6.33% expected; 2.04-fold enrichment; *p* = 1.53 × 10⁻^34^), whereas no enrichment was detected in the central population (5.89% observed vs. 6.33% expected; 0.93-fold; *p* = 0.83). We then asked whether elevated differentiation and reduced nucleotide diversity co-occur disproportionately on chromosome 7a/7b. Windows simultaneously falling in the top 1% of F_st_ and bottom 1% of northern π were significantly enriched on chromosome 7a/7b. Among high F_st_ windows on chromosome 7a/7b in the northern lineage, 48.7% (145/298) also exhibited reduced π, compared to a genome-wide expectation of 22.6%, representing more than a two-fold enrichment (*p* < 2.2 × 10⁻^1^⁶). Notably, many of these regions cluster near the fission breakpoint (Fig. 5).

These results reveal a spatially restricted and lineage-specific pattern of elevated differentiation and reduced nucleotide diversity on the rearranged chromosome. This pattern aligns with theoretical expectations that chromosomal rearrangements can locally suppress recombination, particularly near breakpoints, thereby intensifying linked selection and promoting localized genomic divergence [29].

## Discussion

By integrating demographic modeling with expanded whole-genome sequencing, cytogenetic data, and fine-scale population genomic analyses, we provide strong evidence that the chromosome 7 fission is fixed in northern geladas and likely absent in other gelada populations. We show that the fission is associated with localized increase in F_st_ and reduced nucleotide diversity in the northern population, particularly near the breakpoint. We also provide evidence that the fission likely arose around or after the divergence of northern and central populations approximately 160,000 years ago. Our dataset, including 10 newly generated karyotypes from previously unsampled northern subgroups and new WGS data from 21 southern geladas, offers the most comprehensive view to date of chromosomal variation and population structure in this species. The absence of the fission in central populations, together with its fixation in the north, underscores limited recent gene flow across these groups, a conclusion reinforced by our demographic analyses (Figs. 3 and 4). Together, these data allow us to situate the emergence of the northern fission within the broader demographic and evolutionary processes shaping divergence among gelada populations.

There are three possible branching scenarios for the evolutionary relationships among gelada populations: (1) northern diverging first, leaving central and southern as sisters; (2) central diverging first, leaving northern and southern as sisters; or (3) southern diverging first, leaving northern and central as sisters. Previous evidence has been mixed, with mitochondrial data suggesting that the central population split first [12], whereas nuclear microsatellite data supported an earlier southern divergence [13]. Our expanded WGS dataset supports the southern-first scenario, providing important context for interpreting karyotype evolution (Fig. 3A). Although karyotype data from southern animals remain unavailable, the absence of the fission in central geladas and its fixation in northern individuals, together with the southern-first branching pattern, suggest that the ancestral gelada karyotype most likely carried an intact chromosome 7. Under this reconstruction, the fission would have arisen along the northern lineage after the southern population diverged from the northern–central ancestor, either prior to or following the subsequent split between northern and central populations. An alternative possibility is that the fission was segregating at low frequency in the ancestral gelada population and later became fixed in the northern lineage while being lost in the central lineage (and potentially also in the southern population, whose karyotype remains unknown).

Our demographic analyses also refine the timing of divergence between the northern and central populations. Whereas previous studies estimated the divergence further in the past [15, 17], these analyses relied on visual assessment of MSMC plots without formal demographic modeling, which may have led to overestimation of divergence times. In contrast, we applied two complementary demographic approaches, both of which indicate a more recent divergence (Figs. 3 and 4). We demonstrate that if the fission on chromosome 7 occurred only in the northern population, then its fixation likely occurred in the relatively short evolutionary time of the past ~ 14,000 generations. However, if the fission occurred before the northern and central populations diverged, it cannot be older than the initial divergence of the northern-central lineage from the southern population, which places an upper bound on when the fission could have arisen (~ 34,000 generations).

The fixation of a chromosomal fission in the northern lineage raises the question of its potential role in reproductive isolation. The role of chromosomal rearrangements in speciation has long been debated, and multiple theoretical models have been developed to explain how such rearrangements can contribute to lineage divergence [1–3]. Early chromosomal speciation models emphasized underdominance, in which heterozygotes for rearrangements suffer reduced fertility due to meiotic mispairing and the production of unbalanced gametes [30]. However, these models face the “underdominance paradox”: if heterozygotes have significantly lower fitness, new arrangements should be eliminated early on when they are rare, making their initial spread unlikely in large, randomly mating populations.

More recent theory has emphasized additional mechanisms beyond simple hybrid sterility. Structural rearrangements are predicted to locally suppress recombination, particularly near breakpoints [1]. Reduced recombination can facilitate the accumulation of locally adapted alleles and promote linkage among incompatibility loci. In this framework, rearrangements don’t need to cause hybrid sterility to contribute to divergence; instead, they may promote reproductive isolation through modified recombination dynamics, thus enhancing the effects of selection and drift.

Empirical evidence supports both perspectives. In some systems, chromosomal rearrangements have been shown to cause infertility in hybrids [31]. At the same time, numerous hybrid zones exist between taxa that differ in karyotype [32, 33], demonstrating that chromosomal differences do not inevitably produce complete reproductive isolation. This variation highlights that the strength of chromosomal differences and their effect on isolation varies widely across taxa.

In our system, the presence of F2 and F3 hybrids indicates that reproduction across karyotypic backgrounds is possible. However, the apparent absence of successfully reproducing hybrid males suggests reduced male fertility. This is consistent with Haldane’s rule, which states that in hybrids, the heterogametic sex is more likely to be sterile [34]. These observations are consistent with partial reproductive barriers associated with the chromosomal difference; however additional data, particularly direct measures of hybrid fertility and broader sampling of hybrids, would be necessary to determine if and to what extent the fission contributes to reproductive isolation.

Importantly, our population genomic analyses align closely with expectations that recombination was historically suppressed on rearranged chromosomes in the northern population. Divergence between northern and central geladas is not uniformly distributed across the genome. Chromosomes 7a and 7b show a significant enrichment of highly differentiated regions, and this excess differentiation is accompanied by lineage-specific reductions in nucleotide diversity (π) in the northern population. Windows exhibiting both elevated F_st_ and reduced π are disproportionately concentrated on chromosomes 7a and 7b (Fig. 5), consistent with localized reductions in effective recombination rate on the rearranged chromosome [29]. This suggests that during the period in which the fission was segregating and progressing toward fixation, heterokaryotypic individuals experienced reduced recombination near the breakpoint. This recombination suppression would increase linkage among nearby loci, intensifying the effects of linked selection and drift and amplifying regional differentiation. The absence of comparable enrichment on chromosome 7 in the central population indicates that these patterns are lineage-specific rather than reflecting intrinsic properties of the chromosome itself. These patterns mirror those observed in other systems where structural rearrangements contribute to heterogeneous genomic landscapes of divergence. Rather than acting solely as a discrete postzygotic barrier, the chromosome 7 fission in geladas appears to have shaped divergence by altering recombination dynamics during its rise to fixation.

Our study contributes to growing interest in the evolution of genome architecture in primates, emphasizing the varying role that chromosomal evolution can play in evolutionary relationships. In geladas, we show that a chromosomal fission becomes fixed within one population in a relatively short period of time, demonstrating that substantial structural change can occur over short evolutionary timescales even in lineages with otherwise conserved chromosomal organization. Moreover, the association between the fission and localized shifts in differentiation underscores that structural variants can shape genomic landscapes of divergence. Further comparative work investigating functional consequences associated with the fission will be essential to determine the phenotype and fitness consequences of this rearrangement. By integrating cytogenetics, demographic inference, and whole-genome analyses, our study provides a framework for understanding how chromosomal rearrangements can contribute to lineage divergence and potentially to the early stages of reproductive isolation, consistent with patterns observed across diverse vertebrate and invertebrate systems.

## Conclusions

Our findings contribute to understanding chromosomal evolution in primates by integrating population genomics, cytogenetics, and demographic modeling. We demonstrate that the chromosome 7 fission is fixed in northern geladas and has strongly shaped patterns of genetic divergence and nucleotide diversity across the rearranged chromosomes. In particular, we observe differentiation patterns that are consistent with recombination suppression in the northern population. Demographic modeling indicates that the fission likely arose within the past ~ 160,000 years and provides a refined view of divergence among populations, placing clear temporal bounds on the origin and spread of the rearrangement. Together, these results highlight geladas as a valuable model for studying the role of chromosome evolution in primate diversification and provide a foundation for future work exploring the functional and evolutionary consequences of chromosomal rearrangements in primates.

## Methods

### Data generation

We analyzed whole genome sequencing data from *N* = 149 individuals, representing all three major populations (northern, central, and southern). Our dataset includes 96 wild individuals from the northern population, 3 wild individuals from the central population, 21 wild individuals from the southern population, and 27 captive individuals with confirmed central ancestry (Additional file 2: Table S1). These samples include data previously analyzed by Chiou et al. [15] (NCBI Bioproject: PRJNA470999) [18, 19]. We also incorporated 2 additional southern individuals from Caldon et al. [17] (NCBI Bioproject: PRJEB72259, sample accessions ERR12892801 and ERR12892810) [20].

Of these, 46 newly sampled individuals from the northern population were sequenced for this study, as well as additional central ancestry individuals from NaturZoo Rheine (*N* = 8) and 21 previously unsampled southern individuals. Sample collection and sequencing protocols for northern animals closely followed those reported by Chiou et al. [15]. Briefly, blood samples were drawn from wild individuals during annual capture-and-release immobilization protocols by employing blow dart injections to temporarily sedate the animals. DNA from blood and tissue samples were extracted using Qiagen’s DNeasy Blood and Tissue Kit. Libraries were prepared using the Nextera DNA Library Prep protocol and sequenced on either the Illumina HiSeq X or NovaSeq 6000 platforms.

Captive individuals of central ancestry were sampled during standard veterinary examinations at NatuZoo Rheine. Sequencing was performed at the Cologne Center for Genomics. Unlike previously generated data, WGS data for 21 southern geladas was derived from fecal samples. Fecal DNA was prepared for whole-genome sequencing using the strategy developed by Chiou and Bergey [22].

### Variant calling and filtering

Raw WGS data were combined with previously published datasets [15, 17] and mapped to the gelada reference genome (Tgel_1.0) [15] using BWA-MEM [35] using the same methods described in Chiou et al. [15]. Variants were called using a GATK (v4.6.2.0) [36] implemented in region-parallelized scripts. For each individual, variants were first called independently in GVCF mode using GATK HaplotypeCaller with default model parameters, taking as input duplicate-marked, coordinate-sorted BAM files. Joint genotypes were then generated with GenotypeGVCFs using the GenomicsDB workspace as input. The resulting raw VCF files were compressed, indexed, and validated prior to downstream filtering.

Variant-level hard filtering was performed using GATK VariantFiltration. The following site-level filters were applied: Quality by Depth (QD < 2.0), Mapping Quality (MQ < 40.0), Fisher Strand bias (FS > 60.0), Mapping Quality Rank Sum (MQRankSum < − 12.5), Read Position Rank Sum (ReadPosRankSum < − 8.0), and Strand Odds Ratio (SOR > 3.0). The option –missing-values-evaluate-as-failing was specified so that variants lacking any required annotation were treated as failing.

Following GATK filtering, biallelic single-nucleotide polymorphisms (SNPs) were extracted using bcftools. Only SNPs (-v snps) with exactly two alleles (-m2 -M2) and with a FILTER status of either PASS or. were retained. Indels and multiallelic sites were excluded.

### Population structure analyses

To examine population structure and estimate ancestry proportions, we used two complementary genotype-likelihood-based approaches: a principal component analysis (PCA) implemented in PCAngsd and an unsupervised clustering approach implemented in NGSadmix. As both approaches account for genotype uncertainty, they are particularly robust for low-coverage sequencing data. For both approaches, we used genotype likelihoods from all autosomal chromosomes (chromosomes 1–20, including 7a and 7b) generated using ANGSD v.0.940 [37] in beagle format (arguments: -doGlf 2 -doGeno −4 -doPost 2 -doPlink 2 -doMajorMinor 1 -SNP_pval 1e-6 -doMaf 1), with BAM alignment files mapped to the gelada genome as input [15]. To exclude repetitive elements, we used seqkit v2.5.1 (arguments: -F --only-positive-strand --bed -m 0 -p N) [38] on the repeat-masked version of the gelada genome available through ENSEMBL to identify coordinates of all sites masked by RepeatMasker. These regions were merged using bedtools merge (v2.30.0) [39] to combine adjacent coordinates into repeat regions in BED format. Using the ANGSD results, we output variant coordinates to BED format, then used bedtools subtract (v2.30.0) to identify variants not found in repeat regions. We then used a custom Python script to filter our beagle file generated by ANGSD to include only variants outside of repeat regions. After combining ANGSD results by chromosome, we used a custom Python script to thin variants in high linkage disequilibrium by traversing each chromosome and removing sites within 10,000 bp of each retained site. We then used PCAngsd v1.2 [40] to estimate covariance among individuals and NGSadmix v32 [41] to estimate admixture proportions (arguments: -minMaf 0.05 -P 14). Because the number of populations was not assumed a priori, we explored a range of *K* populations, with *K* varying from 2 to 5 (Additional file 1: Figure S3).

### Genetic diversity

We calculated per-site heterozygosity using folded site-frequency spectrum (SFS) files computed using ANGSD v.0.940 [37]. To first generate site allele frequency likelihood files, we ran ANGSD on single-sample bam files with basic filters to exclude low-quality sites (arguments: -C 50 -minQ 20 -minmapq 30). We then used the realSFS program to compute the folded SFS, from which we computed per-site heterozygosity for each individual.

We identified five southern samples with heterozygosity values exceeding 10^–3^, suggesting that they could include DNA from multiple individuals–liekly due to cross-contamination during non-invasive sampling. We therefore removed these samples prior to testing if heterozygosity differed between the populations using a linear model that controlled for sequencing depth.

### Phylogenetic reconstruction

To infer phylogenetic relationships among populations, we subset the VCF to include two individuals from each population, prioritizing high-coverage wild individuals. The resulting dataset was filtered using VCFtools [46] to retain sites genotyped in at least 80% of individuals and thinned to one SNP every 10 kb to reduce linkage among markers. The filtered VCF was then converted to PHYLIP format using cvf2phylip v2.8 and used to reconstruct a maximum-likelihood phylogeny with IQ-TREE [25].

### Demographic inference

#### SMC++ 

We estimated population split times using SMC++ [28]. A VCF was filtered to only include autosomal regions of the genome, biallelic SNPSs, and to remove missing data before converting to SMC++ format. Split times were estimated using a per generation mutation rate of 5.7 × 10^–9^ (derived from anubis baboons; [45]) and a generation time of 11.67 years [15].

We analyzed three population comparisons using a consistent set of individuals. For the northern–central comparison, we included 11 individuals from each population. For comparisons involving the southern population, we used the same central individuals together with two high-quality southern blood samples and the nine highest-coverage southern fecal samples. Using this same set of individuals, we also conducted a northern–southern comparison.

#### dadi-cli

A two-dimensional, joint neutral site frequency spectrum (SFS) was generated in *dadi-cli* [27] using a VCF file as input. The VCF was filtered using BCFtools [46] to include only putatively neutrally evolving regions > 10 kb from gelada exon sequences. The vcf was also filtered to include only autosomal regions of the genome, biallelic SNPs, and to remove all missing data.

For consistency, the same 11 northern and 11 central individuals used in SMC++ were included in the dadi-cli analyses. We evaluated the fit of our joint SFS data to various demographic models, assuming the same per generation mutation rate and generation time as used in SMC++. We chose to use *dadi-cli* because this program is particularly flexible in model specification and historically successful at inferring demographic history using data from multiple populations. Our analysis focused on two-population models. We chose to consider the following in our evaluation:

1. IM: Isolation-with-migration model with exponential population growth
2. no_mig: Split into two populations, no migration
3. sym_mig: Split into two populations, with symmetric migration
4. no_mig_size: Split with migration, then size change with no migration

For each model, we ran a minimum of 100 replicates, checking for convergence at every 100 additional replicates. The inference was forced to continue in this manner until both parameter estimates and log-likelihood values reached convergence. We ran continuous replicates and convergence checks to ensure that our findings are reliable. Final parameters were chosen based on the highest log-likelihood values (Additional file 2: Table S2).

#### Divergence estimates across the genome

Population genetic parameters were estimated in sliding windows of 50 kb and 100 kb with a 10 kb slide using VCFtools v0.1.14 [47]. We calculated Weir and Cockerham’s F_st_ and within-population nucleotide diversity (π). To minimize the effects from sparsely genotyped regions, we retained only windows containing a minimum number of SNPs. Two SNP-density thresholds were applied independently: ≥ 10 SNPs per window and ≥ 50 SNPs per window. Windows failing to meet the specified minimum SNP threshold were excluded from downstream analyses.

For all statistical analyses, chromosomes 7a and 7b were treated as a single combined unit (hereafter referred to as “chromosome 7a/7b”). Windows from chromosomes 7a and 7b were therefore pooled when calculating summary statistics and when assessing chromosomal enrichment.

To identify regions of elevated differentiation, we defined outlier F_st_ windows as those falling within the top 1% of the empirical genome-wide F_st_ distribution for each window size and SNP threshold combination (Additional file 2: Table S3). Similarly, candidate regions of reduced nucleotide diversity were defined as windows falling within the bottom 1% of the genome-wide π distribution, calculated separately for the northern and central populations (Additional file 2: Tables S4 and S5). All percentile thresholds were computed independently for each window size (50 kb and 100 kb) and each SNP threshold (≥ 10 SNPs, ≥ 50 SNPs).

To test whether outlier windows were disproportionately concentrated on chromosome 7a/7b, we performed binomial tests comparing the observed proportion of outlier windows on chromosome 7a/7b to the expected proportion based on the representation of chromosome 7a/7b windows among all autosomal windows passing quality filters. All statistical analyses were conducted in Rv4.2.2.

#### Metaphase spreads for karyotyping

To determine gelada karyotypes, we prepared metaphase chromosome spreads from cultured fibroblast cells derived from ear and nose biopsy samples. Cells were arrested in mitosis using colcemid solution (10 μl/ml) and harvested by centrifugation at 1200 rpm for 8 min. Following harvest, cells were incubated in a hypotonic solution (0.075 M KCl) for 15 min at 37 °C. Cells were pre-fixed with freshly prepared cold fixative (methanol:acetic acid, 3:1) and centrifuged again at 1200 rpm for 8 min. The pellet was resuspended and fixed with several changes of cold fixative.

Chromosome spreads were prepared by dropping the fixed cell suspension onto pre-cleaned glass slides and air-drying. Slides were aged by baking at 90 °C for 90 min. G-banding was performed by treating slides with a 2.5% trypsin solution (diluted 1:40 in PBS) for 40 s, followed by staining with Giemsa solution. Chromosomes were visualized using a light microscope at 100 × oil immersion magnification. For each individual, a minimum of five non-overlapping, well-spread metaphases were analyzed to determine chromosome number. Karyotypes were confirmed by consistent chromosome counts across multiple spreads (Additional file 1: Figure S4).

## Supplementary Information

Additional file 1. Fig. S1. Inferred phylogeny of zoo hybrids. Fig S2. Genomic distribution of highly differentiated and low-diversity windows. Fig S3. NGSadmix results for K 2-5. Fig S4. Example metaphase spread used in this study.

Additional file 2. Table S1. Gelada samples and associated metadata. Table S2. Dadi-cli estimates for population divergence, effective population size, and migration under each tested model. Table S3. Enrichment of highly differentiated Fst windows on chromosome 7a/7b across window sizes and SNP density thresholds. Table S4. Enrichment of bottom 1% nucleotide diversitywindows in the northern population on chromosome 7a/7b relative to autosomes across window sizes and SNP thresholds. Table S5. Enrichment of bottom 1% nucleotide diversitywindows in the central population on chromosome 7a/7b relative to autosomes across window sizes and SNP thresholds.

## Data Availability

The dataset supporting the conclusions of this article is available in the NCBI repository, (SRA Bioproject PRJNA470999 and PRJEB72259) [18–20]. All code is released under the MIT open-source license and is available at GitHub [48] (https://github.com/brooklynnscott00/gelada-chromosome-evolution) and archived on Zenodo [49].
