# Supplementary material for: Genomic and cytogenetic evidence for divergence and speciation in geladas
Source: Genome Biol. 2026 Jul 22;27:320. doi: 10.1186/s13059-026-04143-6 (PMC13682072; doi:10.1186/s13059-026-04143-6)
Supplement: Supplementary file 1 [file 13059_2026_4143_MOESM1_ESM.docx]

**Fig S1. Inferred phylogeny of zoo hybrids:** Hybrid phylogenies were inferred based on whole-genome ancestry proportions and mitochondrial data from Zinner et al. [[8]](https://paperpile.com/c/MLRFR5/RQHV0). Colors indicate the proportions of northern (orange) and central (green) ancestry in each individual. Both hybrids showed northern mitochondrial haplotypes, represented by the orange arrows. Mitochondrial haplotypes were traced back through the maternal lineage. (A) F3 hybrid with ~13% northern ancestry (B) F2 hybrid with ~ 25 northern ancestry.

**Fig S2. Genomic distribution of highly differentiated (top 1% F_st_) and low-diversity (bottom 1% π) windows:** Windows in the top 1% of the genome-wide F_st_ distribution between northern and central populations are shown in black. Bottom 1% π windows are shown separately for the northern population (orange) and the central population (green).

**Fig S3. NGSadmix results for K 2-5:** Admixture analysis of gelada populations using NGSadmix for K = 2–5. Each bar represents an individual, colored according to the proportion of ancestry assigned to each genetic cluster. Individuals are grouped by population along the x-axis, and the y-axis shows the estimated ancestry proportions. Northern, central, and southern populations group into three distinct ancestral clusters with K = 3. Increasing K leads to increased ancestry groups within the northern population, reflecting refined population structure of the three northern subgroups.

**Fig S4. Example metaphase spread used in this study.**
